# Formative research to adapt a cardiac rehabilitation program to breast cancer survivors: the heart health after cancer treatment (HEART-ACT) study

**DOI:** 10.1186/s40959-024-00228-y

**Published:** 2024-05-17

**Authors:** Alison Chang, Alisa Boyd, Ivan Leung, Evelin Trejo, Niharika Dixit, Jaya Mallidi, Sithu Win, Alexis L. Beatty

**Affiliations:** 1 School of Medicine, University of California, San Francisco, USA; 2 Department of Epidemiology and Biostatistics, University of California, San Francisco, USA; 3 Division of Hematology Oncology, Department of Medicine, University of California, San Francisco, USA; 4 Zuckerberg San Francisco General Hospital, San Francisco, USA; 5 Department of Medicine, University of California, San Francisco, USA; 6 Division of Cardiology, Zuckerberg San Francisco General Hospital, San Francisco, USA

**Keywords:** Breast cancer, Survivorship, Cardio-oncology, Cardiac rehabilitation, Physical activity, Nutrition

## Abstract

**Background:**

Breast cancer survivors are disproportionately at risk for cardiovascular disease; exercise-based interventions may improve cardiovascular health. The objective of this formative research is to better understand the needs of patients and barriers to participation in an adapted cardiac rehabilitation program for diverse breast cancer survivors in an urban safety net setting.

**Methods:**

We recruited 30 participants (10 English-speaking, 10 Spanish-speaking, and 10 Cantonese-speaking) who had received treatment with curative intent for breast cancer from an urban safety net hospital between November 9, 2021, to August 30, 2022. Participants completed surveys and interviews about perspectives on health behaviors and participating in an adapted cardiac rehabilitation program. Interviews were qualitatively analyzed using rapid template analysis with pre-selected constructs from the Theory of Planned Behavior, Unified Theory of Acceptance and Use of Technology, and Consolidated Framework for Implementation Research, as well as emergent codes. We developed a Participant User Journey for a program based on responses and conducted human-centered design sessions with 8 participants to iteratively revise the Participant User Journey.

**Results:**

Among 30 participants, mean age was 56.7 years (standard deviation [SD] 10.2) with 100% female sex assigned at birth; 1 participant withdrew before completing study procedures. Most participants had limited health literacy (18/29, 62%). Mean body mass index was 31.4 (SD 8.3), 21/29 (72%) had blood pressure below 140/90 mmHg, and 12/29 (41%) had blood pressure below 130/80. Mean 6-minute walk distance was 384.9 meters (SD 78.3). The desired benefits of a program included healthy living and prevention of cancer recurrence. Barriers to participation included motivation, social support, transportation, and concerns about exercise safety. Participants emphasized the need for practicality, such as fitting physical activity into daily life and nutrition support, including recipes and shopping lists. Trusted experts and cultural and language concordance were viewed as important aspects of the program.

**Conclusions:**

Through participant interviews and human-centered design sessions, we developed the HEART-ACT program, a 12-week multi-disciplinary program addressing physical activity, nutrition, emotional well-being, cardiovascular risk, survivorship, and other components if indicated (e.g., tobacco cessation). Future research will test the effects of this program on patient-centered outcomes.

**Supplementary Information:**

The online version contains supplementary material available at 10.1186/s40959-024-00228-y.

## Background

Breast cancer survivors are disproportionately at risk for cardiovascular disease compared to similar populations without breast cancer [1–3]. In addition to shared risk factors like tobacco use, obesity, and sedentary lifestyle, the increased risk in breast cancer survivors is compounded by the side effects of cancer treatments, including cardiotoxicity from chemotherapy or targeted therapy, radiation, and hormone-related therapies [4]. Notably, among older breast cancer survivors, cardiovascular disease exceeds breast cancer as the leading cause of mortality [5]. There is an urgent need to develop effective strategies to address cardiovascular risk in breast cancer survivors.

Physical activity is not only associated with a lower risk of cardiovascular disease among breast cancer survivors but also with decreased risk of breast cancer recurrence [4, 6]. Exercise training interventions improve outcomes such as exercise capacity and quality of life in breast cancer survivors [4, 7–9]. In patients with cardiovascular disease, programs that support improving health behaviors more broadly are more effective than interventions with exercise training alone [10]. One such intervention is cardiac rehabilitation (CR), a multi-disciplinary program of exercise training and health behavior counseling that improves outcomes for people with cardiovascular disease [11]. However, it is unknown whether translating the CR model to breast cancer survivors can improve outcomes [12]. Additionally, there are known disparities in the delivery of CR [13]. Thus, adaptation of the CR model to the breast cancer population should seek to understand barriers to participation and address the needs of diverse populations to avoid replicating the disparities seen in the delivery of CR.

The Heart Health After Cancer Treatment (HEART-ACT) study aims to develop and test an adapted CR program for breast cancer survivors to improve cardiovascular disease outcomes and reduce health disparities. Here, the objective of this preliminary research is to better understand the needs of patients and barriers to participation in an adapted CR program for diverse breast cancer survivors in an urban safety net setting. This formative research resulted in the design of a CR program tailored to meet the needs of breast cancer survivors.

## Methods

### Study design

This mixed methods study collected quantitative data from questionnaires and body measurements and qualitative data from semi-structured interviews with participants in two phases. The first phase included questionnaires, body measurements, and semi-structured interviews with participants about attitudes and needs regarding participating in an exercise and health behavior counseling program. Based on participant responses in the first phase, we generated a Participant Journey Map (Supplemental Material). The Participant Journey Map was initially refined based on feedback from a Community Advisory Board including cardiovascular disease and breast cancer patients and breast cancer advocates. In the second phase, we conducted human-centered design sessions with participants to elicit feedback on the Participant Journey Map.

### Participants

We consecutively screened participants scheduled for in-person visits at the breast cancer clinic at Zuckerberg San Francisco General Hospital, an urban safety net hospital in San Francisco CA. We recruited 30 participants meeting eligibility criteria from November 9, 2021, to August 30, 2022. Inclusion criteria were age 18 or older, diagnosis of breast cancer within the past 5 years, treated with curative intent, able to communicate in English, Spanish, or Cantonese, and able to consent for self. Participants were excluded if they had chemotherapy, radiation, or surgery within 3 months, or if life expectancy was less than 1 year. All participants provided written informed consent for participation.

### Measurements

We collected demographic information from participants including age, sex at birth, gender identity, race/ethnicity, highest education attained, and subjective socioeconomic status [14]. We collected breast cancer history and medical history. Participants had blood pressure measured in duplicate using an automated blood pressure cuff (Omron Series 10). Weight, height, and waist-to-hip ratio were measured by study staff. A six-minute walk test was conducted following a standard protocol [15]. Participants completed standard questionnaires including: Patient-Reported Outcomes Measurement Information System (PROMIS) Global Health (Physical and Mental subscales, v1.2) [16], PROMIS Cognition Function – Short Form (v 2.0) [17], Chronic Disease Self-Efficacy [18], Food Security 2-question screening [19], Rapid Eating and Activity Assessment for Participants – Short Form (REAP-S) [20], and International Physical Activity Questionnaire (IPAQ) Short Form [21]. Health literacy was assessed with a single question about confidence in filling out medical forms [22, 23]. Medication adherence was assessed by asking the participant how many times they missed medications in the last 7 days [24]. Participants completed custom questionnaires on medical conditions, substance use, and digital literacy. If translated versions of standard questionnaires were available, translated versions were used. Otherwise, questionnaires were translated from English into Spanish and Cantonese. Participants were shown questionnaires in their preferred language.

Quantitative data were collected and managed using REDCap electronic data capture tools hosted at UCSF.

### Semi-structured Interviews

We conducted individual semi-structured interviews with participants in their preferred language using an interview guide (Supplemental Material) by trained bilingual research staff (IL, ET). The interview guide was developed in consultation with our Community Advisory Board. Interviews were audio recorded and transcribed. Transcripts were translated to English. Translated transcripts were reviewed by bilingual research staff for content and meaning with revisions made for inaccurate transcription.

### Qualitative analysis

We performed rapid template analysis [25] of interviews based on Theory of Planned Behavior [26], concepts from the Consolidated Framework for Implementation Research [27], and the Unified Theory of Acceptance and Use of Technology [28, 29], as well as concepts of health literacy, digital literacy, and health disparities (template in Supplemental Material). Emergent codes were generated for concepts not well captured by pre-specified codes. Two study team members (AC and ABo) independently reviewed interviews and transcripts and coded responses based on the template, then met to achieve concordance. Representative quotations in the domains were selected for description of the findings.

### Human-centered design sessions

Human-centered design sessions on the Participant Journey Map were conducted in a convenience sample of 8 participants who had previously participated in study measurements and interviews (3 Spanish-speaking, 2 Cantonese-speaking, 3 English-speaking) between August 2022 and December 2022. Research staff (IL, ET) presented the Participant Journey Map to participants. While reviewing the Participant Journey Map, research staff recorded participant reactions and comments. In addition, research staff elicited comments from participants with prompts such as: “What is your reaction to …?”, “What else would be helpful?”, “Any likes or dislikes?”, “Anything missing?”, “What stands out to you?”. Records from design sessions were reviewed by two independent study team members (ABo and ABe), with responses categorized as “reinforcing” or “improvement” by element of the Participant Journey Map. Human-centered design output was reviewed with the research staff conducting the human-centered design sessions for content and meaning. Iterative revisions were made to the Participant Journey Map based on participant feedback over the course of the 8 sessions.

### Statistical analysis

Descriptive statistics, including mean, standard deviation and proportions were calculated for measures. For measures with skewed data, median and interquartile range were calculated. For PROMIS Physical Health, Mental Health, and Cognition Function, the Health Measures Scoring Service was used to calculate T-scores and standard errors, which were used to calculate 95% confidence intervals. For IPAQ measures, MET-minutes/week for walking was calculated as minutes/week of walking times 3.3, MET-minutes/week for moderate activity was calculated as minutes/week of moderate activity times 4, and MET-minutes/week for vigorous activity was calculated as minutes/week of vigorous activity times 8. All analyses were performed with Stata version 17 or 18.

## Results

Among 30 participants, mean age was 56.7 years (standard deviation [SD] 10.2 years) with 100% female sex assigned at birth (Table 1). Participants were diagnosed with breast cancer a median of 2.8 years prior to enrollment (interquartile range 1.5 – 3.9 years).The study included 10 English-speaking, 10 Spanish-speaking, and 10 Cantonese-speaking participants. One participant withdrew before completing all study questionnaires and the interview.

**Table 1:** Participant characteristics

**Characteristic**	*N*=30
Age (years), mean ± standard deviation (SD)	56.7 ± 10.2
Female sex assigned at birth, n (%)	30 (100%)
Female gender identity, n (%)	30 (100%)
Race/ethnicity*, n (%)	
* Black or African American*	2 (7%)
* White*	4 (13%)
* Asian*	12 (40%)
* Native Hawaiian or Pacific Islander*	1 (3%)
* American Indian or Alaska Native*	0 (0%)
* Hispanic, Latino, or Spanish*	11 (37%)
* Other*	8 (27%)
* Don’t know*	2 (7%)
Subjective socioeconomic status, mean ± SD (1 = lowest, 10 = highest)	5.6 ± 2.4
Highest level of education, n (%)	
* No formal schooling*	1 (3%)
* Did not graduate high school*	7 (23%)
* High school diploma or equivalency*	11 (37%)
* Associate degree*	4 (13%)
* Some college but did not graduate*	3 (10%)
* Bachelor’s degree*	3 (10%)
* Prefer not to state*	1 (3%)
Primary language, n(%)	
* English*	10 (33%)
* Spanish*	10 (33%)
* Cantonese*	10 (33%)
Types of breast cancer therapy^a^, n (%)	
* Surgery*	29 (97%)
* Chemotherapy*	14 (47%)
* Radiation*	21 (70%)
* Targeted therapy*	1 (3%)
* Endocrine/hormone therapy*	27 (90%)
Years since diagnosis, mean ± SD	2.8 ± 1.5
Medical conditions, n (%)	
* Hypertension*	7 (23%)
* Diabetes*	6 (20%)
* Congestive heart failure*	1 (3%)
* Sleep apnea*	5 (17%)
* Anemia or other blood disorder (not including leukemia or lymphoma)*	1 (3%)
Current tobacco use, n (%)	2 (7%)

^a^Participant could select more than one answer

Most participants had limited health literacy – reporting confidence in filling out medical forms as “Not at all,” “A little,” or “Somewhat” (18/29, 62%) (Table 2). Most participants 24/29 (83%) had a smartphone and 26/29 (90%) participants had at least one device capable of accessing the internet (smartphone, tablet, or computer). A minority of participants used the health system’s online portal (9/29, 31%) (Table 2).

**Table 2:** Health and digital literacy

**Item**	*N*=29*
**Health Literacy, n (%)**	
How confident are you filling out medical forms?	
* Not at all*	4 (14%)
* A little*	3 (10%)
* Somewhat*	11 (38%)
* Quite a bit*	4 (14%)
* Extremely*	7 (24%)
**Digital Literacy, n (%)**	
Which of these devices do you have?^a^	
* Smart phone*	24 (83%)
* Fitness tracker or smartwatch*	2 (7%)
* Tablet*	9 (31%)
* Computer or laptop*	7 (24%)
Online portal use (MyChart)	9 (31%)
Video conferencing use	18 (62%)
Video conferencing confidence (*N*=18)	
* Not confident at all*	1 (5%)
* Somewhat not confident*	3 (17%)
* Neutral*	3 (17%)
* Somewhat confident*	6 (33%)
* Very confident*	5 (28%)
How do you access the Internet?*	
* Smartphone*	20 (67%)
* Tablet*	5 (17%)
* Home computer or laptop*	2 (7%)
* Work computer or laptop*	1 (3%)
* Community computer*	2 (7%)
* Other*	1 (3%)
* I don’t access the Internet*	7 (23%)
Use internet for health information (*N*=22)	17 (77%)
Confidence in finding health information on internet (*N*=17)	
* Not confident at all*	2 (12%)
* Somewhat not confident*	2 (12%)
* Neutral*	2 (12%)
* Somewhat confident*	7 (41%)
* Very confident*	4 (23%)
Concern about cost of internet access	
* Not concerned at all*	6 (21%)
* Somewhat not concerned*	2 (7%)
* Neutral*	5 (17%)
* Somewhat concerned*	5 (17%)
* Very concerned*	5 (17%)
* Not applicable*	6 (21%)
Concern about online privacy	
* Not concerned at all*	2 (7%)
* Somewhat not concerned*	1 (3%)
* Neutral*	2 (7%)
* Somewhat concerned*	8 (28%)
* Very concerned*	10 (34%)
* Not applicable*	6 (21%)

^*^ out of *N*=29 participants, except where noted

Mean BMI was 31.4 (SD 8.3), 21/29 (72%) had blood pressure below 140/90mmHg, and 12/29 (41%) had blood pressure below 130/80 (Table 3). Mean six-minute walk distance was 384.9 meters (SD 78.3). Participants reported moderate physical health, mental health, cognitive function, and chronic disease self-efficacy. Though 10/29 (34%) reported some food insecurity, average diet was moderately healthy (mean REAP-S 30, SD 4.8, scale 13-39 [best]). In the last 7 days, 7/29 (24%) had missed doses of medications. Physical activity was mostly walking, with a median of 180 minutes of walking per week. No physical activity was reported in 7/29 (24%) of participants.

**Table 3:** Health measures and health status

**Characteristics**	*N* = 29
Body mass index (kg/m^2^), mean ± standard deviation (SD)	31.4 ± 8.3
Systolic blood pressure (mmHg) mean ± SD	122.4 ± 14.9
Diastolic blood pressure (mmHg), mean ± SD	81.0 ± 10.5
Blood pressure <140/90 mmHg, n (%)	21 (72%)
Blood pressure <130/80 mmHg, n (%)	12 (41%)
Blood pressure <120/80 mmHg, n (%)	11 (38%)
Waist to hip ratio, mean ± SD	0.9 ± 0.1
Six-minute walk distance (meters), mean ± SD	384.9 ± 78.3
Physical Health T-score, mean (95% confidence interval [CI]) (23.4-63.3 [best])	41 (33, 49)
Mental Health T-score, mean (95%CI) (25.8-64.6 [best])	44 (37, 51)
Cognitive Function T-score, mean (95%CI) (24.99-61.13 [best])	47 (41, 53)
Chronic Disease Self-Efficacy, mean ± SD (1 - 60 [totally confident])	38.6 ± 13.9
Hunger Vital Signs, n (%)	
Within the past 12 months we worried whether our food would run out before we got money to buy more (often or sometimes).	10 (34%)
Within the past 12 months the food we bought just didn't last and we didn't have money to get more (often or sometimes)	9 (31%)
Rapid Eating Assessment Score, mean ± SD (13-39 [best])	30 ± 4.8
Missed at least 1 medication in last 7 days, n (%)	7 (24%)
International Physical Activity Questionnaire, median (interquartile range)	
Walking, MET-minutes/week	594 (0, 1386)
Moderate physical activity, MET-minutes/week	0 (0, 360)
Vigorous physical activity, MET-minutes/week	0 (0, 0)
Total MET-mins/week	954 (297, 1848)

### Qualitative Results

#### Attitudes

For most people, physical activity is important for maintaining a sense of happiness, strength, independence, as well as for its positive impact on mental health and overall sense of well-being. Some people stated the importance of physical activities that can be easily incorporated into daily routines, like walking or housework. However, barriers to physical activity include fear of physical strain, limitations due to cancer or other health conditions (e.g. requiring a wheelchair) and a subsequent sense of discouragement or frustration, and lack of motivation or time (see Supplemental Qualitative.

“It’s good, of course, if you can do it, but you have to see if you have that much strength. Sometimes I am afraid I cannot hold on if I do too much physical activity.”

“I think I’m active enough. I keep doing exercises, going to work, going to the library and reading, doing housework or going shopping every day.”

“Well, I don't think I'm doing enough. I don't think I'm physical enough to where it's making a difference in my life. ...So I need to find that motivation to do it because I know, at the end of the day, even if I'm eating right, to keep a healthy heart, to keep healthy legs and blood flow, that I need to be more active. And I just don't have the motivation to be active.”

#### Beliefs

While there are some people who recognize the benefits of physical activity on having heart disease, they conceded that exercise should be in moderation or individually personalized, as excessively strenuous exertion may be harmful as well. There was also confusion on what constitutes adequate exercise to have this benefit on their health.

“As long as such physical exercise is not too strenuous, then it’s beneficial to the heart, and this is my personal feeling.”

“I think if you have cancer, then it’s your destiny.”

“I knew that it reduces the risk of having a heart attack or cancer coming back, yes. If you stay in bed or you stay at home and you don't exercise at all, your metabolism, your blood is not flowing well.”

“I don't know because a lot of people say that they have friends with cancer who had to change their habits completely. They have to be more active, they have to stop eating certain things, they have to start eating healthier, and they have to lose weight because if they don't, that's bad for their health and there's more risk of recurrence. I don't know if that's true because that's what they tell me. I don't know if that's how it is.”

#### Subjective norms

Many participants identified family, friends, and in some cases, even pets, as pivotal sources of support and care during their experience with cancer. Additionally, patient support groups and health navigators emerged as influential groups. The nature of this support encompassed diverse forms such as companionship through walking together, preparing meals, providing encouragement, accompanying individuals to medical appointments, facilitating access to dietary resources, among other types of assistance. The medical team was also commonly mentioned as a source of trust and advice.

“My oncologist, … she's been great from the beginning. I had a navigator through general hospital… was great in helping me with resources, especially when I started chemo and got to the point where I wasn't able to work. The [community program] helped me after chemo, when I was tired and didn't feel so well, they would deliver prepared meals for me.”

“it was more like just a group of ladies, kind of like a reflection group… So it was fun to just kind of get back to normalcy because it seems like when you're going through treatment, you kind of get knocked off of your normal routine and your normal behaviors and you got to kind of just be more focused and geared to healing yourself and getting healthy again.”

“No. My family didn’t take care of me, and I had to cook meals for them. Yeah, I also cooked meals on the day of my surgery.”

“I'm out every day because I have a dog… I make my rounds, that's how I call it, to the shops, that I make friends with the owners or the people who work there..”

#### Perceived behavioral control

People named the following barriers to perceived control of their ability to exercise or engage in healthier lifestyles: lack of prior exercise habits, limited mobility (e.g. wheelchair requiring an in-home care provider), reduced physical strength and increased fatigue owing to cancer treatments or other health conditions (e.g. substance abuse), fear of physical exertion and its impact on health, low motivation, limited time due to competing interests (e.g., work), and an unsafe environment limiting exercise in the community. Meanwhile, factors that would help facilitate people’s perceived ability to exercise include access to gym equipment, incorporation of activities into daily life and routine, and for some, religion.

“I used to be very careful about my diet, but I was nervous and under a lot of pressure at work because I was doing two jobs, one full-time and one part-time. I was the guarantor for the immigration of one of my relatives, so I was under lots of pressure. Anyway, I was very careful with my diet. However, I was pretty busy at work, I didn’t have time…”

“I'm in a wheelchair because of an accident… I have limited mobility”

“God gave me another chance at life, he's going to give it to me and I'm going to move on. That's how I started doing my exercises, always walking, although sometimes in the morning I would say, "I'm not going to get up, I feel bad physically and mentally." I was like, "No, no, no, that's not going to bring me down. God, help me."”

“Sometimes when I take the medication, especially for the first year after the surgery, the side effects were so strong that I was exhausted all the time.”

“ I didn't have time to exercise or anything; I worked a lot and that was enough. As a janitor, my work was very hard. It was seven hours on my feet walking.”

“I think if I had a schedule… I think it would motivate me better than me trying to find something to do on my own.”

#### Attitude toward technology

Most people who chose to participate in this study voiced a preference for in-person sessions as it allows for better communication, engagement with the speaker, and guidance during group exercises or activities. Though generally people expressed understanding about using video-conferencing during the pandemic and valued its convenience, barriers to its use included privacy concerns and the potential for distraction.

“Yeah, I've heard of [video-conferencing]. I hate that. ... I'm looking at what's behind you on the screen and forgetting all about what you're talking … that is totally nosy”

“if we're going to be in person, I would like to have an activity where all the people who are going to be there can participate with the speaker; an activity to remain physically active, as you say. If it's on [video-conferencing], we could also have an activity, but it would be different because everyone would be at their home.”

#### Performance expectancy

Some people expressed that they currently use technology for health and fitness for very specific purposes (e.g., tracking distance or exercise minutes, music for dancing, video conferencing calls). Some people commented on the convenience and flexibility of using video-conferencing for visits. Barriers to use of technology for visits included concerns about clarity of communication, privacy, and connection with peers.

“It’s likely that it’s not as clear on the phone as it could be face-to-face"

“If you did all that, it would be phenomenal, because there would be like a guide that I would follow. I’d like it by [video-conferencing]. I’d like to do it at home so I don’t have to move anywhere, look for a parking space, for running late.”

“I would prefer in-person, because that way you can look at the person, and learn more and understand better.”

#### Effort expectancy and facilitating conditions

There are widely varying comfort levels with video-conferencing, including people who are well-versed in navigating video-conferencing and those who express unfamiliarity or ongoing challenges. Such challenges include forgetting or not knowing how to use video-conferencing and connectivity issues, though a few people mentioned that they were amenable to being taught and proposed solutions such as having a guidebook or receiving live assistance from a family member.

“Yes. I do know it. The thing is, to be honest, I'm not very good with technology, it's a bit difficult for me, but maybe if it's at a time when my daughter is here, they can help me, or maybe I can learn before the day of the session. I imagine it shouldn't be that difficult, I just haven't been interested, really.”

“So the facilitator was just on video conferencing alone. Nobody could come in and she didn't know what the reason was. So that needs to be definitely good playbook how to do these things. Of course, we'd have good playbook...”

#### Design quality and packaging

Many people offered a broad variety of suggestions to market the intervention, ranging from providing incentives such as gift cards or food, to having flyers distributed in the community to emphasize the health benefits of the program. Some viewed doctors as important figures for promoting the program, with an additional emphasis on having people from diverse backgrounds involved in program design. A sense of belonging (including language concordance), use of humor and fun/positivity, and incorporation of goal-setting were proposed as motivating factors for engagement in the program.

“ I think the speaker is quite important… Whoever that can deliver all the message in a fun way.”

“When you go to a group meeting, it’s good to have a defined goal on what you’re going to do.”

“Have a diverse range of ethnicities involved in the planning process… Humor got so many women through the different support groups and sometimes it was a bit [culturally tailored].”

“I think it’s important to stay optimistic, cheerful and strong to live on.”

#### Cost

One individual spoke to the importance of financial accessibility and the potential barriers that costly food or medications and a restrictive work schedule can pose to implementing the intervention.

“During these sessions, they should also offer women who are struggling with cancer places where they can, maybe not get it for free, but buy these items at a lower price. They could set a place where we could go and show a card or our ID itself and buy stuff for us. I don’t know if that already exists.”

#### Patient needs and resources

Exercise equipment and accessible exercises for people with disabilities (e.g. requiring a wheelchair) were highlighted by several individuals as needs that should be known and prioritized by the organization.

“Yoga sitting down is also good for people who are older. So they don't really feel like lying on the floor and then struggling to get up.”

“Depending on what exercise I do. I need [exercise equipment], and a pair of comfortable shoes to better protect my feet and a loose sportswear for running. Those are the most basic things I need.”

#### Individual stage of change

Some individuals commented on already feeling back to “normal” and confident, having already received adequate support and education after completing cancer treatment. On the other hand, some voiced an ongoing process of reckoning with the physical, psychological, and spiritual elements of their breast cancer journey, with a few expressing that they need to build a stronger sense of motivation and responsibility for their health.

“For me, when you are done and you don't really need to learn anything because I didn't have any problems after the treatment. My life is back to normal. But maybe some people are stressed or depressed about the whole thing, and then maybe they need help. But I really don't.”

“I am right now processing what my body has been through mentally... I had a very serious sickness and it was a big deal. I am very grateful for my team... Right now, I feel very well taken care of. I do have some physical limitations because of side effects from treatment, including arthritis, because I believe it's the anastrozole that I'm taking post-cancer treatment that leads to arthritis, but I'm also have a physical therapist that's helping me with that.”

“What would I need? I would just honestly need the motivation. Once I get motivated to do something, once I get focused on something, I'm focused on that, but I just need something to get me motivated. So like I said, a program, a schedule, something for me to follow.”

#### Language

Language concordance was deemed important for participant engagement and comfort. Some people proposed bilingual speakers as a solution for participants who require different languages.

“I think doing it in different languages. I personally would feel comfortable knowing that there's someone speaking my language, and if I have a question or if there's anything I need, I know there's that person.”

#### Health literacy

People discussed gathering health information from the Internet (e.g. Google, YouTube, Yahoo News) or from television. Others mentioned the library or support groups as valuable sources of information.

“There are definitely times where being able to look something up on the internet kind of can ease your mind between appointments or when you can't speak to a doctor.”

“I joined multiple support groups and some of them are about fitness and weight loss and that means encouraging each other even when we are not rushing out there and doing marathons…”

#### Digital literacy

Among the interviewees, the ability to use technology for health varies. Some reported that they did not know how to use apps to help manage their health or could only access MyChart with assistance. Others reported being more frequent users of mobile health applications, ranging from the Health app on Apple Watch for tracking steps to a Chinese app called TouTiao for health-related news and information. Barriers to digital health use include concerns about misinformation and privacy, as well as issues with ease of use and internet connection.

“I'm a little cautious, since we have all these misinformation floating around”

“I learnt it through the Internet. I don’t ask about the website. What I love to read is TouTiao. I can’t figure out Facebook that you mentioned, because I can’t read English. What I search is about Chinese network and read some science stuff.”

“This App for example, on the one hand, helps me keep track of where I’ve been and how many miles I’ve traveled each day, which is good. On the other hand, I think it violates my privacy and forces to know where I’ve been, which I don’t feel comfortable with.”

“There's an app called MyChart or something, I don't remember what's it called. I installed it on my phone during the pandemic to see my COVID test results, but I'm very bad remembering passwords. I installed it, but after I opened it a couple of times, I pressed a button where I had to type my passwords again.”

#### Timing and Duration of program

Sentiments around timing and duration of the program were mixed, with some participants opposed to night sessions.

“I don't like nighttime. I like during the day and the afternoon, morning, and every day's okay… I don't like to be outside at night by myself.”

“I think one hour would be convenient because-- Well, I don't know if everyone will have obligations to fulfill or if they have time.”

“It could be any day, but preferably in the afternoon because I can't leave the house until I get one of my children to come stay with their father. I can take him, but getting him on the bus and everything, is hard for both of us.”

#### Mixed genders and diagnoses

The main barriers to having mixed-gender sessions are concerns around the comfort level of participants in sharing personal experiences and the belief that some cancers, such as breast cancer, can raise issues that are uniquely specific to women. However, the majority of people expressed openness to having mixed-gender sessions.

The interviewees expressed varying preferences regarding sessions with different diagnoses. Some preferred to have sessions with only patients who have the same type of cancer, particularly breast cancer, due to a shared sense of understanding. Others were open to having sessions with any cancer patient, but not with patients who have non-cancer health conditions because the experiences may be too varied. There were also some individuals who were open to all.

#### Nutrition

There is a strong desire to learn about nutrition and to understand what to eat, particularly to prevent cancer recurrence. Barriers to current nutrition knowledge include confusion and conflicting information, such as the difference between what doctors recommend and what people hear from others in their communities. Some patients raised concerns regarding the financial implications of adopting a healthy diet and the variable accessibility to affordable produce. Facilitators include education from experts with professional guidance on what to eat, how to cook (“how to” recipes, methods to best obtain nutrients from food), and meal planning, delivered in a culturally tailored way.

“I would love to learn more about nutrition. I do my best to eat right. And I know I had a pretty good idea of what is bad for us and what isn't. And I've seen the list of cancer causing foods, donuts, French fries, bacon, cookies. And I enjoy cooking. So it's just exploring recipes, techniques of healthy food that would be enjoyable. And learning how to feed myself properly. That's essential to healing.”

“In some cases, we could be taught to make our own meals, for those of us who work. You know that we sometimes need to leave the house at 6:00 AM and get ready for work. Those meals need to be prepared the day before or something like that… The hardest part… is the financial situation, that we often don’t have the means to buy them.”

#### Stress management

Some people acknowledged the need to manage stress and its sources, as well as the desire to learn practical ways to handle daily stressors. Common sources of stress were the COVID-19 pandemic, persisting struggles with illness, and the multiple demands of work and caregiving. Here, they proposed specific ideas such as seeking support from others, learning breathing exercises, and thinking positively. Others expressed less enthusiasm for this topic, as they voiced that stress management was not personally relevant or needed.

“For pressure management, I know a lot of people have such issue, and they suffer from huge psychological pressure. I don’t know whether it’s because I’m an optimistic person, I have managed the pressure very well, so there is no need for me to hear about it. ... “

“We can learn how to make peace with ourselves and get rid of idea of being a patient like “I’m sick, it’s tough for me, or I have to take pills every day”. It gives us positive energy, getting rid of the idea of being a patient.”

“We all get stressed at home, especially if you have kids. Truth be told, I'm always stressed out with everything that's happening to me. I get very stressed out because I can't do my normal activities. I need to go to work, and I can't go to work. I need to do home chores, such as vacuum the house, but now it's very difficult for me because of the weight and the movement of the vacuum, my arm doesn't let me. As for my hand, I can't open a bottle, I have to ask someone else to open it for me. When it comes to slicing vegetables, I can start slicing them, but eventually, I have to stop because of the pain. When I'm scrambling an egg, I have to stop because it hurts.”

#### Mindfulness

People expressed varying levels of familiarity and interest in mindfulness.

“I have heard that it has some effect, but I have little interest in it.”

“Mindfulness is something I'm working on.”

#### Survivorship

The main themes that emerged were the importance of self-care and mental resilience; prevention and fear of cancer recurrence; “moving forward”; recovery that extends beyond cancer treatment; and the impact of positive thinking. Other topics that were raised include the long-term side effects of treatments, such as cognitive impairment or lymphedema, and the role of religion in helping some people build mental fortitude throughout their cancer experience. People proposed oncologists and other professional experts as trusted speakers, expressing the hope that the support would be ongoing and long-term.

“Survivorship has been motivating.”

“For me, being a cancer survivor has been something hard, but I keep a positive outlook. I always thank God because He gave me a second chance at life and to be mentally positive.”

“I’m very interested in learning how to protect myself so it doesn’t come back.”

“Well now I want survivorship to include something about cognitive impairment because I didn't understand how much that was going to change…”

#### Sex, intimacy and body image

While most people did not express interest in this topic, learning points included confidence and self-esteem issues related to body image, the impact of treatment and medication on intimacy and desire, pain with sex, and communication issues with partners. One participant highlighted her concern regarding the potential impact of makeup products on cancer recurrence. Some participants expressed that this topic would not be personally relevant as they are not sexually active, and others described embarrassment or discomfort with discussing sexual health questions with their doctor as another potential barrier.

“There are days when I get in the shower and I just want to cry because I don't recognize the body and I might cry right now. And it's just, I'm ultimately grateful that I'm alive and they were able to remove the disease from my body, but you know, it's still painful.”

“My sexual appetite was totally extinguished.”

#### Sleep

People valued learning how to improve sleep habits, understand factors affecting sleep quality, and obtain benefits of good sleep. Some also expressed interest in learning about how meditation or medication can improve sleep quality, in addition to creating a calm sleep environment. Current barriers to good, uninterrupted sleep include racing thoughts, stress, insomnia, and even environmental factors like pets.

“Sleep, I actually would benefit from that because during my treatment, and even now with some of the medications that I'm on, sleep is a tough one for me because there'll be times where I can't sleep at night or I have difficulty falling asleep or my sleep is easily broken. And that could also contributed to some of the fatigue I'm having because I'm not sleeping or getting enough sleep or maybe getting too much sleep.”

#### Alcohol/tobacco/cannabis

People expressed widely varying opinions about alcohol/tobacco/cannabis use. Some viewed these substances as harmful and irrelevant to their cancer treatment or lacked sufficient knowledge about them. One person expressed interest in learning how to avoid these substances, especially for patients who struggle with addiction. On the other hand, several others found them (particularly cannabis) helpful or at least expressed interest for evidence-based guidance about their health effects, benefits, and ways to use them safely.

“Well, everybody should be informed about what they can do to you. What type of effects they have to you which you can prevent, how you can and just try not to.”

#### Heart conditions risk factors and medications

There is a clear interest in learning about heart disease risk factors and medications, with a need for education particularly on its relationship to breast cancer. People expressed interest in learning about medication side effects, hypertension and the hereditary nature of heart disease given a strong family history in some patients.

“This is a big topic and yes, I'm very curious about what the treatments and additional medications do to our body besides fight the cancer. I've had mugas, which I didn't know what those were before cancer and based on the mugas, my heart is getting weaker and that's scary.”

“Yes, I would be very interested in knowing more about heart disease related to people who have had cancer, what the risk of heart disease is and knowing more about that topic. My sister even had a heart attack at an early age. My mother and father died of a heart attack. I would like to know more about that topic.”

#### Additional topics of interest

Topics of interest for additional classes included: beauty and appearance, including tips on styling hair after chemotherapy; financial assistance; maintaining a positive mindset to support recovery; possibilities of cancer recurrence; connecting with other people who have kids; the harms of substance abuse and its relationship to cancer; the psychological and emotional effects of dealing with cancer (including chemo fog).

#### Program staff

For program staff, people prefer expert professionals and specialists in their topics who are also language concordant. There is a strong emphasis on reliability, expertise, and connection with the participants, through a sense of understanding and perhaps even personal experience (speakers who themselves are cancer survivors). While most patients did not have a preference for male or female speakers, some patients expressed greater comfort and relatability in asking questions to a female speaker for certain topics.

“It would be nice to have a nutritionist. As for the heart, a professional who specialized in that area; and as for the medications, someone who also specializes in that area so that they can properly explain to us about it. As for self-esteem, it would be good to have a professional because it's not the same if I'd tell you like, "You're going to be okay. You're a beautiful person, you're this and that," than having someone who knows about these things who could give you a piece of advice.”

#### Session setting: home or community

People expressed interest in doing group exercises in community settings, such as the YMCA. Here, the main facilitators are social support, motivation, greater enjoyability and better air quality. People also voiced a preference for a dedicated exercise space, if they were to be in the community. There was also acknowledgement that the at-home setting allows for convenience amid different schedules, with some suggesting that the pandemic may have shifted people’s preferences for exercising at home due to risk of COVID-19 exposure.

“I would enjoy at a facility because it gets me out of the house. I'll be honest, sometimes when I'm in the comfort of home, I'm not as energetic. I don't know. I just feel like I'll be more lazy at home. ...So if I can get out the house and do it, I think it would be better for me.”

“I used to go out a lot before, but not since the outbreak, because at the senior center, you always get in contact with the elderly. I have some sense of protecting myself.”

### Human-centered design

The program was designed to resemble a cardiac rehabilitation program, meet the needs of participants, and address barriers to participation (Table 4). The elements of the program were presented to participants in human-centered design sessions and iterative changes were made to refine the program design.

**Table 4:** Program elements to address patient needs and barriers

**Need/Barrier**	**Program Element**
Motivation	Individualized treatment plans based on participant’s goals with coaching from a health promotion specialist.
Fear of overdoing it with exercise, physical limitations	Individualized exercise prescription and opportunities for supervised exercise.
Transportation, safety of in-person evening sessions	Provide home exercise equipment to participants (peddler, weights, resistance bands).
Practical nutrition advice	Will include a nutritionist and provide food demonstrations, recipes, and shopping lists.
Culturally tailored foods	Will provide food options across multiple different cultural styles.
Cardiovascular risk education	Cardiovascular risk education a core component of the program.
Mental health stigma	Will include elements on emotional well-being and mindfulness, presented as emotional well-being rather than mental health. Emphasize optimism.
Topics specific to cancer survivorship	Will emphasize prevention of cancer recurrence, include topics such as pain management and other survivorship topics tailored to the individual.
Support from other cancer survivors	Group sessions for education and exercise.
Support from family/friends	Family/friends can join individual sessions.
Support from clinicians	Regular sessions with program health promotion specialists. The program will regularly communicate with the participant’s clinical team.
Varying preferences for mode of delivery	Will tailor the mode of delivery (e.g., in-person or telehealth) to patient preference for individual sessions.
Language concordance	Will have bilingual staff delivering the intervention in the participant’s preferred language.

Participant feedback from human-centered design sessions was reinforcing with regard to a 12-week, individualized, multi-disciplinary program including exercise, nutrition, emotional well-being, cardiovascular risk, survivorship, and other components as needed. Participants supported the individualized nature of the program and patient-centered goal-setting. Participants highlighted benefits including prevention of cancer recurrence and learning more about healthy living. Nutrition components stood out to participants, including having access to a dietician, presenting culturally-tailored foods, and focusing on practical aspects such as recipes and shopping lists. Participants' responses varied with regard to having a scheduled gym time at ZSFG for exercise. Some cited barriers related to transportation and safety, particularly regarding evening sessions, and alternatively proposed that the program could provide exercise equipment for use at home by the participants. Suggestions for improvement included changing “mental wellness” to “emotional well-being” for greater cultural acceptability, especially amongst Spanish-speaking participants. A suggestion was made to add pain management to potential topics. Participants desired the ability to bring family members to individual sessions. Communication between the program and their regular clinicians was requested by participants. Recruitment suggestions included placing fliers in the infusion center. A desire for continued connection and support after the initial 12 weeks of the program was expressed by several participants. Participants selected the study name of HEART-ACT, representing Heart Health After Cancer Treatment, from among several choices.

## Discussion

**Fig. 1:**
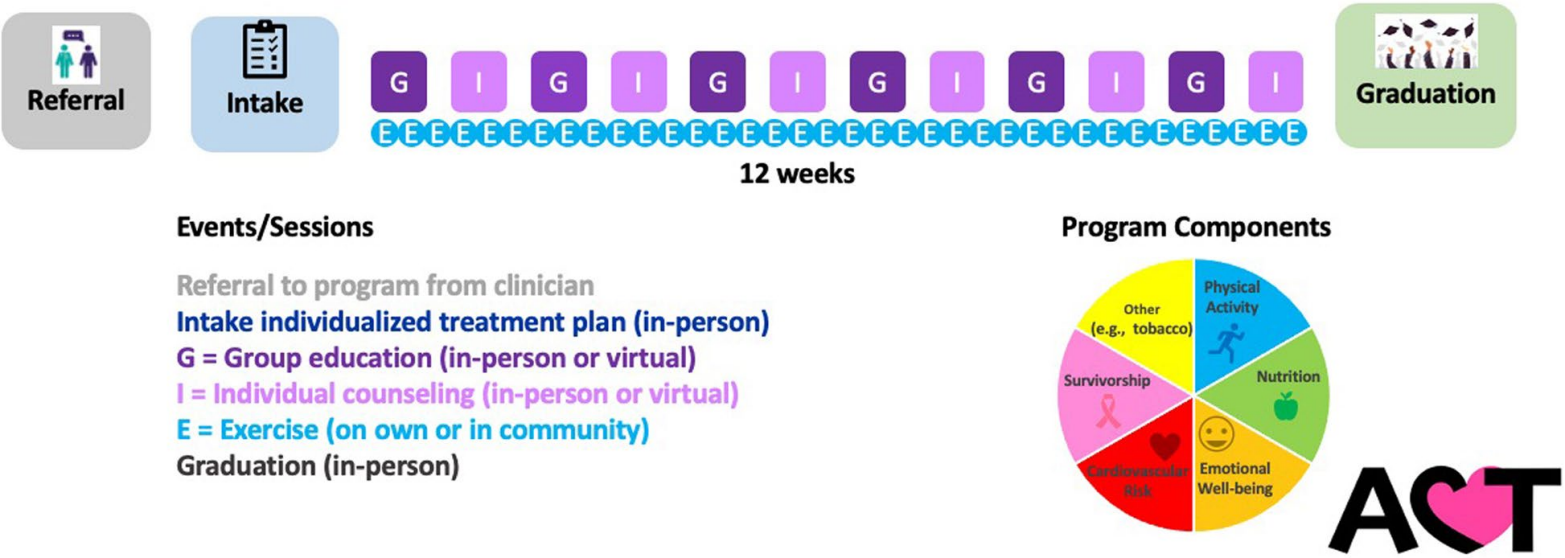
HEART-ACT Program. Components of the HEART-ACT program: physical activity, nutrition, emotional well-being, cardiovascular risk, survivorship, and other components if indicated (e.g., tobacco cessation). The program is designed as a 12-week program. At the intake session a nurse or exercise physiologist conducts an intake interview and assessments (e.g., questionnaires, blood pressure, six-minute walk test) and generates an individualized treatment plan with the participant. Participants will have an individualized exercise plan that will gradually progress towards meeting or exceeding United States physical activity guideline recommendations of 150 minutes per week of moderate to vigorous activity and 2 episodes per week of strength training [30]. Participants alternate between group education sessions (which include physical activity, nutrition, and other topics) and individual sessions for 12 weeks. At the conclusion of the program, participants repeat intake assessments and celebrate at a graduation

This formative research revealed that breast cancer survivors desired additional support for adopting healthy behaviors. Expected benefits included healthy living and prevention of cancer recurrence. Participants emphasized the need for practicality, such as such as integrating physical activity into daily life and seeking nutrition support that includes access to recipes and shopping lists. Learning from trusted experts and cultural and language concordance were viewed as important aspects of the program. Based on participant input, we ultimately developed the HEART-ACT program (Fig. 1), a 12-week multi-disciplinary program addressing physical activity, nutrition, emotional well-being, cardiovascular risk, survivorship, and other components if indicated (e.g., tobacco cessation).

Recent scholarship and advocacy have proposed the creation of cardio-oncology rehabilitation programs [4, 31]. Many studies have focused on exercise training, either during cancer treatment or in survivorship, generally demonstrating improvement in quality of life and exercise capacity [7–9, 32, 33]. Breast cancer survivors participating in CR programs have been observed to have significant improvements in health outcomes [34]. A recent randomized trial also went beyond simply providing exercise training to provide a multi-component, multi-disciplinary CR intervention, resulting in improvement in exercise capacity and quality of life [35]. Though some previous studies have focused on breast cancer patients with known cardiovascular disease, our findings suggest that even patients without diagnosed cardiovascular disease have high risk for future cardiovascular events. For example, the six-minute walk distance in our sample was roughly similar to the six-minute walk distance for women participating in CR in general [36]. This suggests that this is a population with low functional exercise capacity, even if they do not have diagnosed cardiovascular disease.

However, many questions remain about cardiovascular risk reduction interventions in patients with breast cancer. In addition to understanding long-term effects of these interventions in larger populations, there is a need to understand how to equitably implement programs. Though it is attractive to build upon the CR model as a means to deliver cardiovascular risk reduction interventions in breast cancer survivors, it is important to build interventions that address the needs of diverse patients. Though our findings are similar to past qualitative analyses of similar programs with regard to expected benefits of interventions and the need for support, our findings support additional dimensions to these analyses [37]. Especially notable findings are the desire for very practical approaches to physical activity and nutrition, as well as language and cultural concordance in delivery of the program.

The implementation of cardiovascular risk reduction programs in breast cancer patients should not be viewed as a replacement for other ongoing survivorship care. Indeed, participants highlighted a desire for the program to be integrated into their ongoing longitudinal care. With many patients reporting ongoing issues with physical or cognitive limitations related to breast cancer treatment, each patient’s individualized assessment and treatment plan should consider the potential need for additional rehabilitation services beyond a multi-disciplinary health behavior intervention.

With evidence building for adapting the CR model to breast cancer survivors and attention to meeting the needs of diverse populations in studying and implementing programs, there is opportunity for improving cardiovascular risk in breast cancer patients. Future research should test the efficacy of adapted CR interventions on patient-centered outcomes, including long-term effects, in diverse populations and settings. This research should also examine the influential factors contributing to program implementation, thereby informing the translation of research findings into practice.

### Limitations

Though we recruited diverse participants with three preferred languages, because this study is conducted in an urban safety net environment, the results may not be generalizable to other settings or populations. The sample size of 30 participants is not large, but we believe that we achieved saturation of major concepts by the end of the analysis. For some concepts, there was no consensus among participants (e.g., timing and composition of sessions), suggesting that additional individualization of the program may be required to meet individual participant needs.

## Conclusions

Through participant interviews and human-centered design sessions, we developed the HEART-ACT program (Fig. 1), a 12-week multi-disciplinary program addressing physical activity, nutrition, emotional well-being, cardiovascular risk, survivorship, and other components if indicated (e.g., tobacco cessation). Future research will test the effect of this program on patient-centered outcomes.

### Supplementary Information

**Supplementary Material 1. **

## Data Availability

No datasets were generated or analysed during the current study.

## Abbreviations

CI: Confidence Interval
CR: Cardiac Rehabilitation
SD: Standard Deviation
