# Supplementary material for: Formative research to adapt a cardiac rehabilitation program to breast cancer survivors: the heart health after cancer treatment (HEART-ACT) study
Source: Cardiooncology. 2024 May 17;10:28. doi: 10.1186/s40959-024-00228-y (PMC11100255; doi:10.1186/s40959-024-00228-y)
Supplement: Supplementary file 1 — Supplementary Material 1. [file 40959_2024_228_MOESM1_ESM.pdf]

Supplemental Material

Interview Guide

Analysis Template

Participant User Journey

The Better Heart Study
PARTICIPANT INTERVIEW GUIDE

Introduction (written informed consent already obtained):

Thank you for agreeing to talk with me today! I would like to ask you a few questions that will help us create a new program for physical activity, health, and wellness for women with breast cancer.

My name is <*insert name*>, and I am a team member for this project. I will be facilitating our discussion, which will take between 40-60 minutes of your time. I am interested in all of your ideas, comments, and suggestions. There are no right or wrong answers. All comments, both positive and negative, are welcomed. Your opinion is important, and I want you to feel comfortable in saying what you really think.

The project team and I want to make sure that we accurately capture all of your ideas; therefore, we would like to ask for your permission to record our discussion. However, we will keep this recording confidential and for use by people involved with the research only. Before we get started, I just want to remind you that your participation in this interview is completely voluntary. You should feel free at anytime to let me know if you need a break or if you do not want to talk about a certain topic or answer a question. You can also let me know if you want to stop the interview at any time.

Any questions before we begin? *[Now start the recording.]* *Start interview.*

Introduction and verbal consent (written informed consent not already obtained):

Hello, my name is - <*insert name*> from the University of California, San Francisco and I am a team member for this project. Alexis Beatty is the researcher in charge of this study. Thank you for agreeing to talk with me today. As we have discussed, you are being asked to take part in this study because you have a history of breast cancer and receive care at Zuckerberg San Francisco General Hospital (ZSFG). Today we are consenting you for the interview part of the study. We will ask you for signed written informed consent when you come to the clinic to complete the other parts of the study.

If you agree to this interview, I would like to ask you a few questions that will help us create a new program for physical activity, health, and wellness for women with breast cancer. The interview will take between 40-60 minutes of your time. There will be no direct benefit to you for participating in this interview, but the information you provide will help us create a new program that we hope will improve heart health in breast cancer survivors in the future. I am interested in all of your ideas, comments, and suggestions. There are no right or wrong answers. All comments, both positive and negative, are welcomed. Your opinion is important, and I want you to feel comfortable in saying what you really think.

The project team and I also want to make sure that we accurately capture all of your ideas; therefore, we would like to ask for your permission to record our discussion. However, we will keep this recording confidential and for use by people involved with the research only. Before we get started, I just want to remind you that your participation in this interview is completely optional. You should feel free at anytime to let me know if you need a break or if you do not want to talk about a certain topic or answer a question. You can also let me know if you want to stop the interview at any time.

Do you have any questions before we begin?

Would you like to participate in this interview? [Now start the recording.]
If person consents, document in study record. Start interview.

If you have questions about this study in the future, you can contact Dr. Alexis Beatty at 415-502-6191 or BetterHeart@ucsf.edu. If you have questions or concerns about your rights as a research participant, you can call the UCSF Institutional Review Board at 415-476-1814.
Interview QUESTIONS (~40-60 minutes)

Instructions for facilitator: These questions do not have to be read verbatim. They are topics to be used as a guide for steering the conversation. Generic prompts: If responses are limited or require clarification, probes may be used to elicit more detailed responses. Probes should use words or phrases presented by the participant using one of the following formats:

Tell me more about ________.
You mentioned ________; what did you mean by that?
Give me an example of _________?
Tell me about a time when _________?

Note: *Ideally all questions/topics will be covered during the interview. For guidance, the starred (*) questions below indicate the questions of greatest importance ("priority questions"), and where probing for additional information/richer descriptions should be prioritized. The starred questions should also be prioritized if the participant has limited time or refuses to complete the entire interview.*

I. Cancer

Now, can you briefly tell me about what your life and health were like before your cancer diagnosis?

Tell me a little bit about your experiences with cancer care.

- Where are you currently in your cancer care?
- Who helps you with your cancer care? How do you feel about this?
  - PROBE: Do you get help from the clinic?
  - PROBE: Do you get help from family, friends, and community?

*What do you do now to take care of your physical health?

*What role, if any, does physical activity play in your life (physical activity can include exercise, leisure activities, housework, yardwork, and activities you do at work/occupation)?

- PROBE: What role did physical activity play in your life before cancer diagnosis?
- PROBE: Did this change during or after cancer treatment? How?
- PROBE: What types of activities do you do?
- PROBE: What helps you to be active?
- PROBE: What keeps you from being active?
- PROBE: Why is physical activity important (or not) to you?

How do you think physical activity affects your risk of breast cancer recurrence?

How do you think physical activity affects your risk of heart disease?

Did you know that physical activity reduces your risk of breast cancer recurrence and your risk of heart disease?
PROBE: Have you talked with your primary care physician (PCP) or oncologist about this?

II. Proposed program

Now, I would like to talk about how the program might be structured. First, I'd love to hear what you are most interested in learning about/having the opportunity to do as part of a health and wellness program?

- *Can you list the top three things you would like to learn about/discuss in a group? Out of the three you mentioned, what would be your number one pick and why?

- *We are considering holding sessions, on the following topics:
  - Physical activity
  - Nutrition
  - Stress management
  - Mindfulness
  - Survivorship
  - Sex & Intimacy (alt. body image and intimacy)
  - Sleep
  - Alcohol/Tobacco/Cannabis
  - Heart condition risk factors and medications
- *What do you think of these topics? If we were to hold one session on each, what would you want to make sure is included?
- What else would you be interested in, that isn't on this list?
- Who/what kind of speakers would you like to have during these sessions?
- How do you feel about having male speakers/presenters versus female speakers/presenters?
- *How do you feel about committing to 12 weekly sessions?
  - Are there specific days and times that would work best for you?
- *We want this program to be enjoyable and comfortable for people from many different backgrounds. What do you think we should do to ensure that this is the case?
  - Would you want to be meeting with the same or other patients each session?
- *How would you feel about doing sessions by phone, video or both? Can you talk about any experiences you've had with clinic visits by phone or video?
    - PROBE for challenges: What worked? What didn't work? etc.
    - PROBE: Are you familiar with the app called Zoom?
- How do you feel about talking about yourself in the group?
- How would you feel about receiving exercise training?
  - [if talks about previous experience] PROBE: What did you like/dislike about that experience?
- *How would you feel about exercising with a group?
  - What if the group had mixed gender?
  - What if the group had people with multiple different health conditions (such as heart or lung disease)? (probe about only women who have had breast cancer?)
- *How would you feel about exercising at home or in your community (YMCA or senior center)?
  - What would you need to start exercising?
  - Where would prefer to exercise?
- Would you be interested in working with a Dietician? Pharmacist? Mental Health Care provider?
  - [if talks about previous experience] PROBE: Tell me more about that?
  - If so, on what topics?
- Have you used mobile apps to help with your health?
  - What did you like/dislike about using the app?
- What should we call this program?

III. Thank you/wrap up

"Thanks for sharing many helpful ideas with us, and now we are coming near the end of our discussion, my last questions for you are…"

- What other thoughts or ideas would you like to share?
- Any thoughts on how to engage people to participate in this program?
- What questions do you have?

Participant ID:
Date of Review:
Reviewer:
General Reflection on Interview:

Construct	Exemplar Quote(s)		Reflection
**Theory of Planned Behavior – use to code for general health behaviors**			
**Attitudes**<br><br>*Thoughts or feelings about a behavior (quotes related to a specific behavior e.g., role physical activity plays in life)*			
**Beliefs**<br><br>*Acceptance of statements as truths or facts (other quotes that may not be about a specific behavior, may include thoughts on how PA affects risk of heart disease and recurrence of breast cancer)*			
**Subjective Norms**<br><br>*Perception that an important person supports a behavior (quotes related to what doctor and/or family member thinks)*			
**Perceived Behavioral Control**<br><br>*Perception of the ability to perform a behavior*			
**Behavioral Intention**<br><br>*Degree to which the subject has a plan to perform the behavior*			

**Unified Theory of Acceptance and Use of Technology – use to code for behaviors related to using technology include telephone, Zoom, mobile apps, wearables**			
**Attitude toward technology**<br>*General thoughts or feelings about the technology*			
**Performance expectancy**<br>*Degree to which using a technology will provide benefits to consumers in performing certain activities*			
**Effort expectancy**<br>*Degree of ease associated with consumers' use of technology*			
**Facilitating conditions**<br>*Consumers' perceptions of the resources and support available to perform a behavior*	.		
**Social influence**<br>*Extent to which consumers perceive that important others believe they should use a particular technology*			
**Habit**<br>*Extent to which an individual believes the behavior to be automatic*			
**Price value**<br>*Consumers' cognitive tradeoff between the perceived benefits of the applications and the monetary cost for using them*			
**Hedonic motivation**<br>*The fun or pleasure derived from using a technology*			
**Technology use intention**<br>*Degree to which the subject has a plan to use a technology*			

Consolidated Framework for Implementation Research			
**Intervention Characteristics**			
**Intervention source**<br><br>*Perception of key stakeholders about whether the intervention is externally or internally developed*			
**Relative advantage**<br><br>*Stakeholders' perception of the advantage of implementing the intervention versus an alternative solution*			
**Design quality and packaging**<br><br>*Perceived excellence in how the intervention is bundled, presented, and assembled (quotes related to structure and delivery of the program e.g., in-person vs. video/Zoom)*			
**Cost**<br><br>*Costs of the intervention and costs associated with implementing the intervention including investment, supply, and opportunity costs*			
**Outer Setting**			
**Patient needs and resources**<br><br>*The extent to which patient needs, as well as barriers and facilitators to meet those needs, are accurately known and prioritized by the organization*			

Characteristics of Individuals			
**Knowledge and beliefs about the intervention**<br>*Individuals' attitudes toward and value placed on the intervention as well as familiarity with facts, truths, and principles related to the intervention*			
**Self-efficacy**<br>*Individual belief in their own capabilities to execute courses of action to achieve implementation goals*			
**Individual stage of change**<br>*Characterization of the phase an individual is in, as he or she progresses toward skilled, enthusiastic, and sustained use of the intervention*			
**Other personal attributes**<br>*A broad construct to include other personal traits such as tolerance of ambiguity, intellectual ability, motivation, values, competence, capacity, and learning style*			
**Other**			
**Language**<br>*Language concordance*			
**Health Literacy**<br>*Ability and challenges related to reading and understanding health information and navigating health care system*			
**Digital Health Literacy**<br>*Ability and challenges related to using technology for health*			

**Emergent**			
Duration of program			
Mixed genders			
Mixed diagnoses			
Physical activity			
Nutrition			
Stress management			
Mindfulness			
Survivorship			
Sex & Intimacy (alt. body image and intimacy)			
Sleep			
Alcohol/Tobacco/Cannabis			
Heart condition risk factors and medications			
Sessions include the same or different people each time			
Intervention name			
Topics of interest			
Facilitators/speakers			
Session setting: home or community			

Overall Map

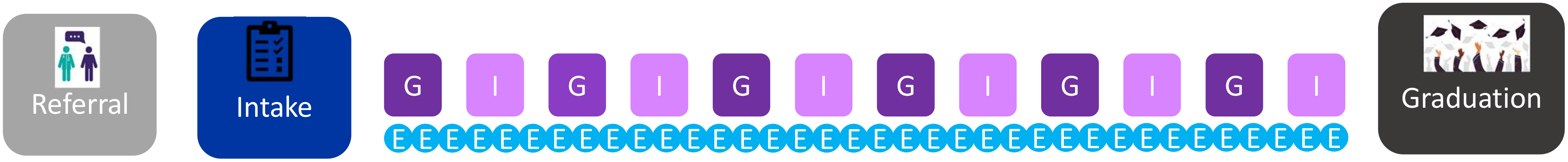

12 weeks

Components of Program

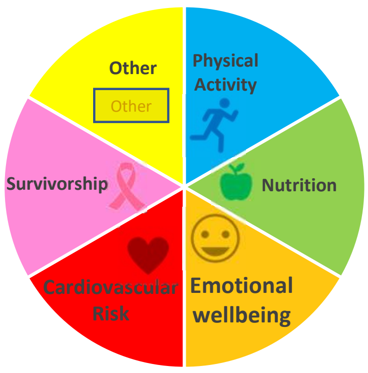

Referral
Intake (in-person)
G = Group Education (in-person)
I = Individual Counseling (in-person or virtual)
E = Exercise (on own or in community)
Graduation (in-person)

Program Components

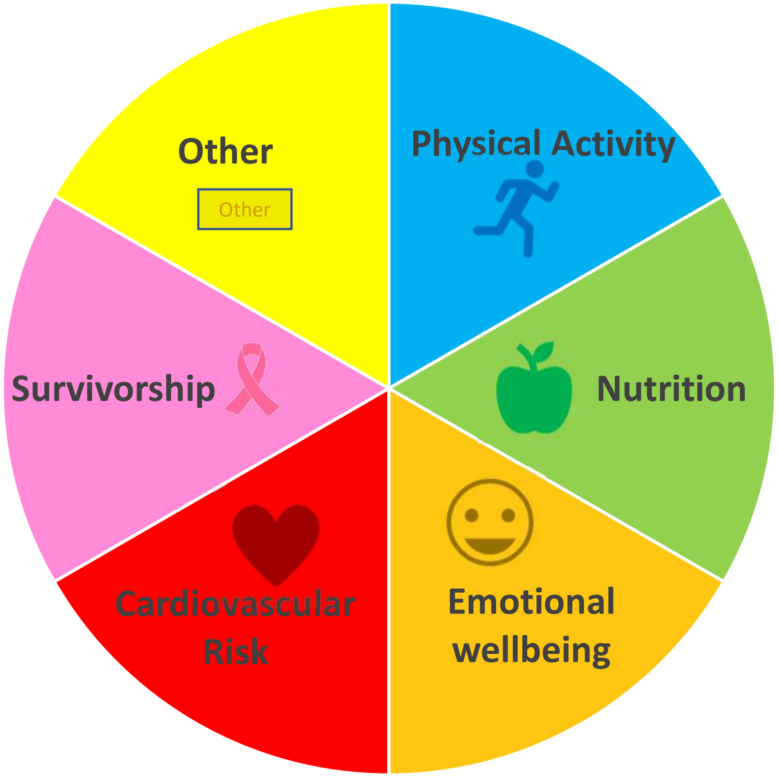

Overall Map

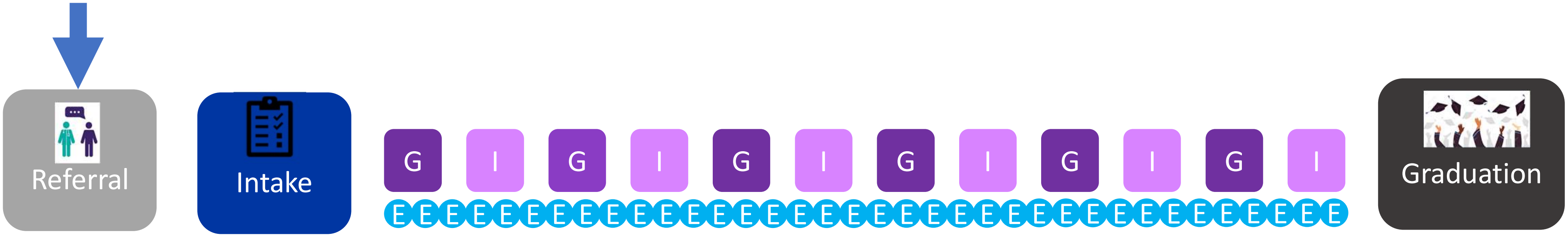

12 weeks

Referral
Intake (in-person)
G = Group Education (in-person)
I = Individual Counseling (in-person or virtual)
E = Exercise (on own or in community)
Graduation (in-person)

Components of Program

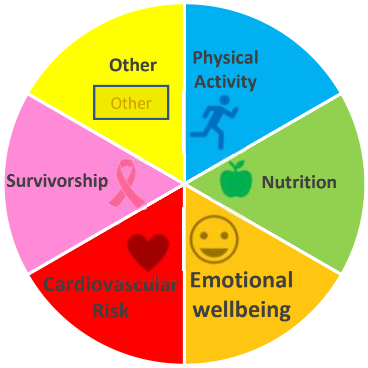

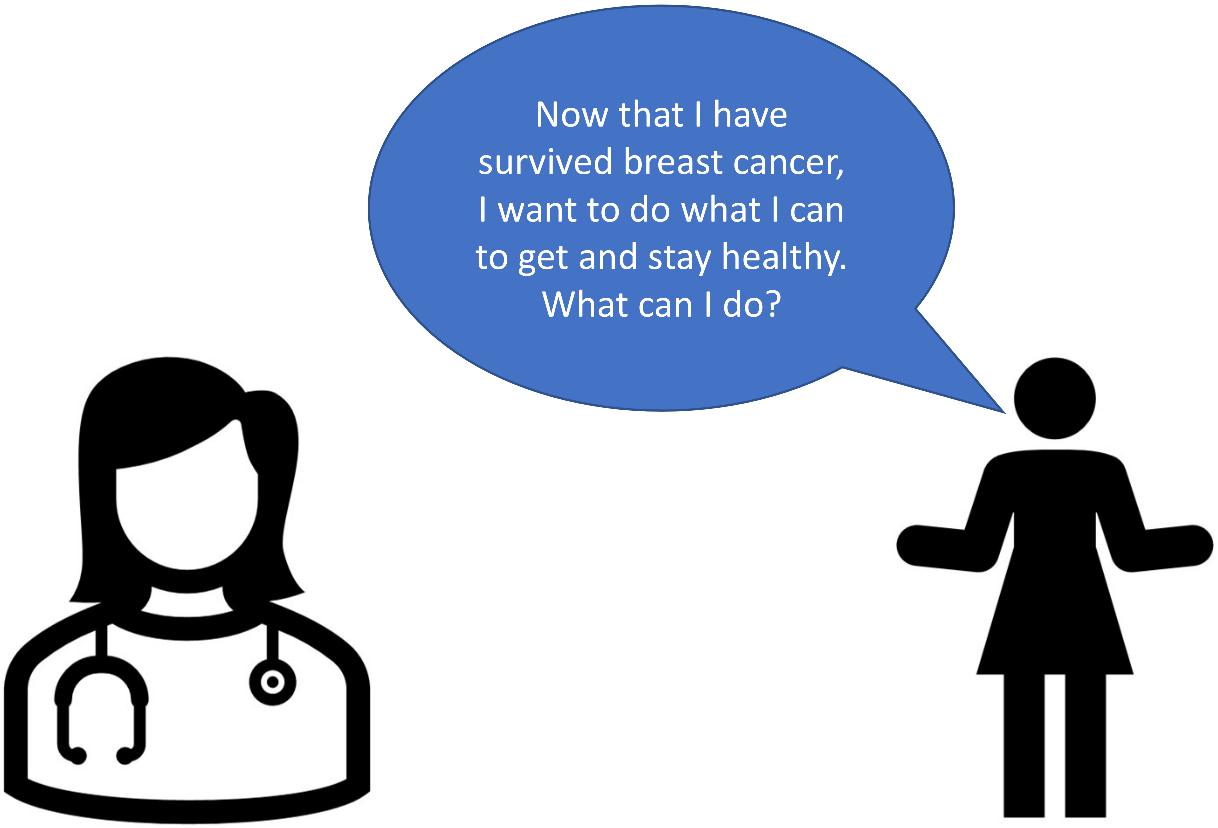
Now that I have survived breast cancer, I want to do what I can to get and stay healthy. What can I do?

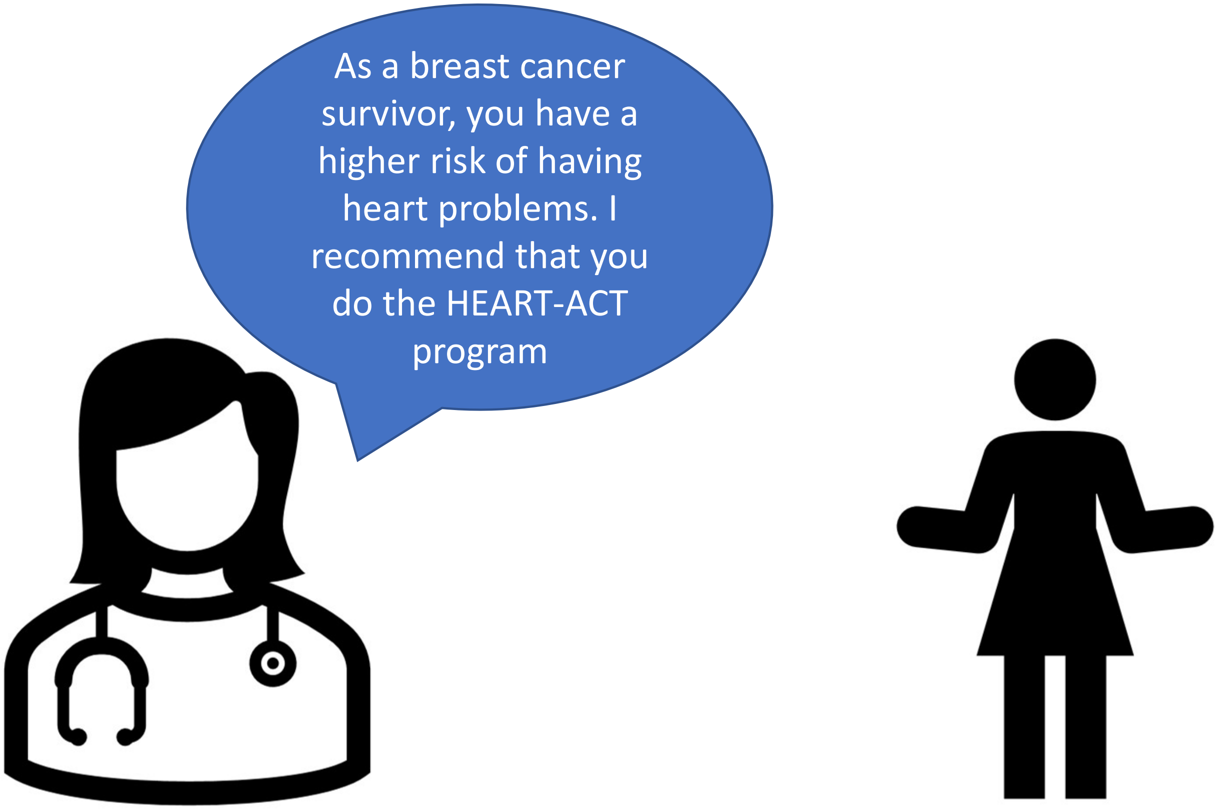
As a breast cancer survivor, you have a higher risk of having heart problems. I recommend that you do the HEART-ACT program

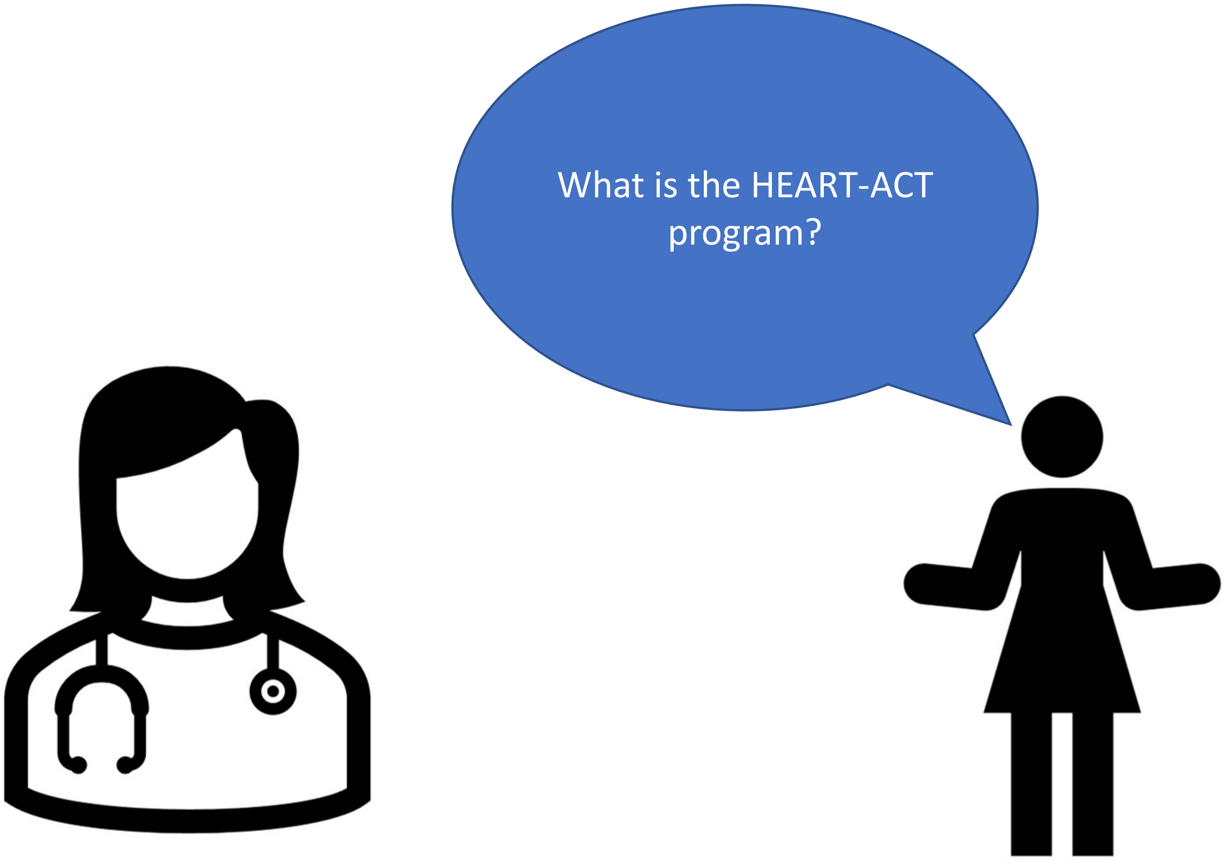
What is the HEART-ACT program?

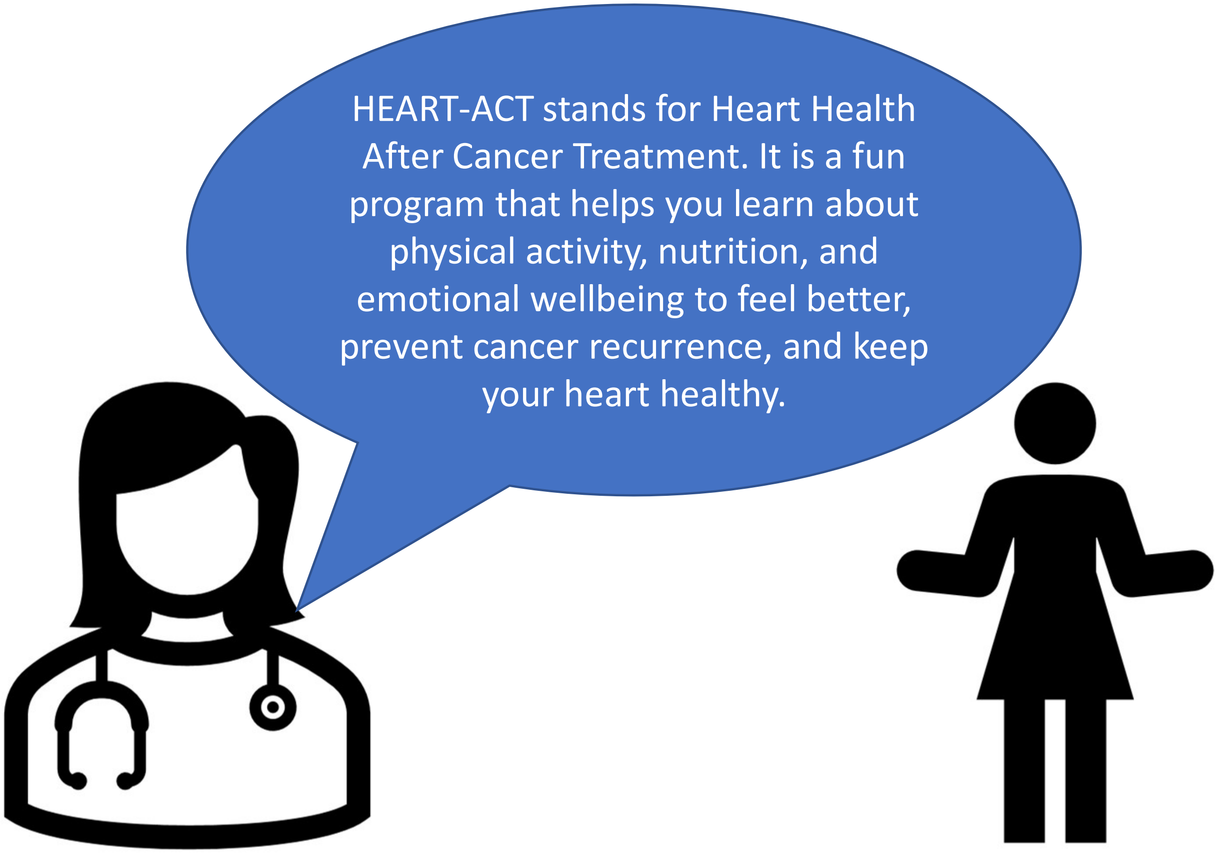
HEART-ACT stands for Heart Health After Cancer Treatment. It is a fun program that helps you learn about physical activity, nutrition, and emotional wellbeing to feel better, prevent cancer recurrence, and keep your heart healthy.

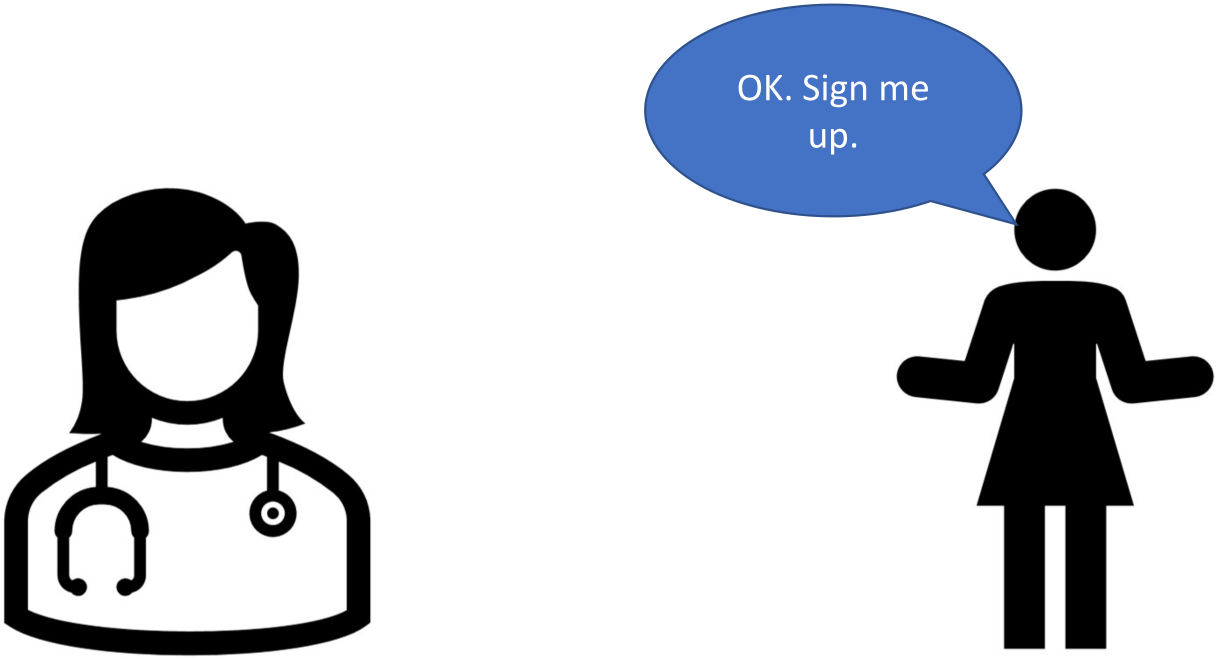
OK. Sign me up.

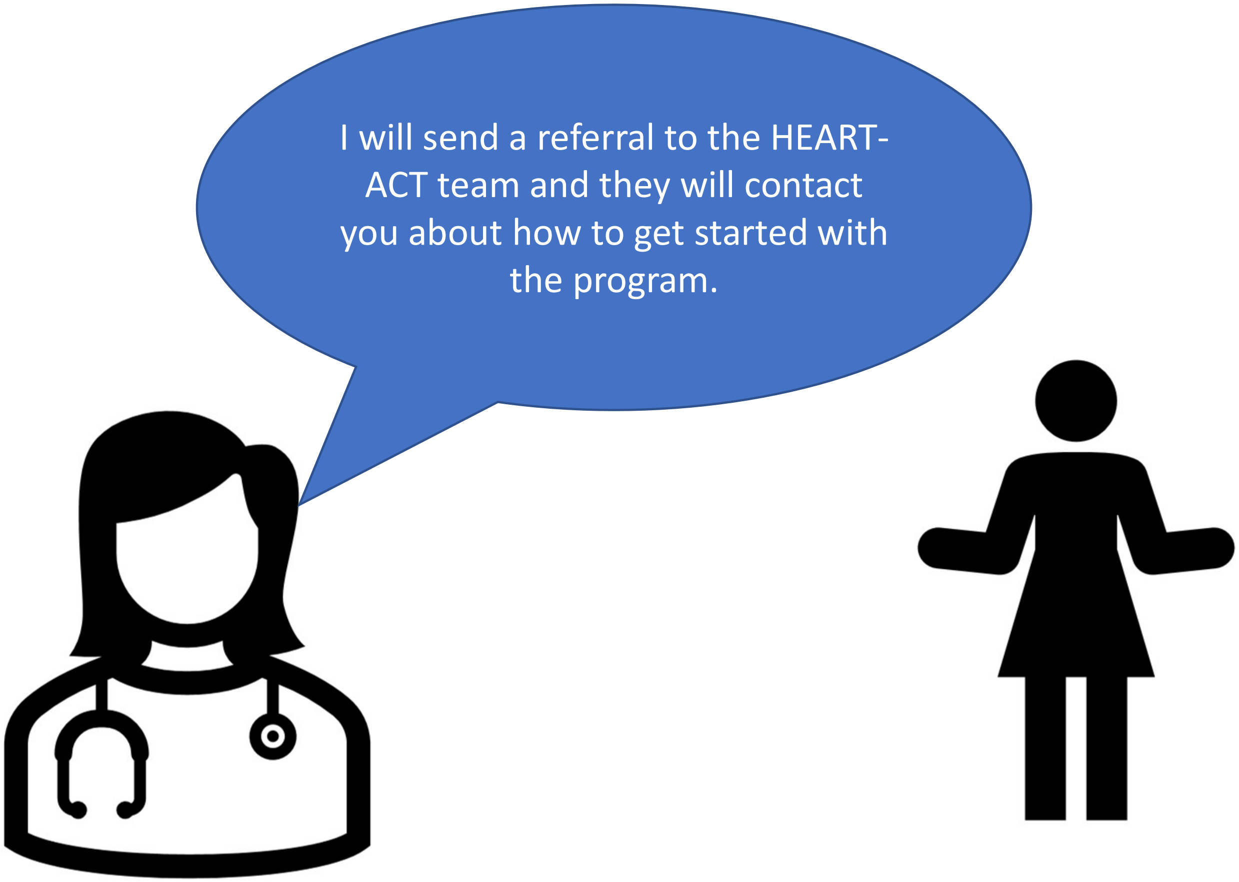
I will send a referral to the HEART-ACT team and they will contact you about how to get started with the program.

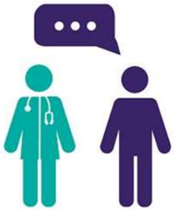

Referral

Primary = oncologist at a survivorship visit

Alternative

- Other health professionals
  - PCP
  - Nurse
  - Navigator
- Flyers
- Community organizations
- Word of mouth

Overall Map

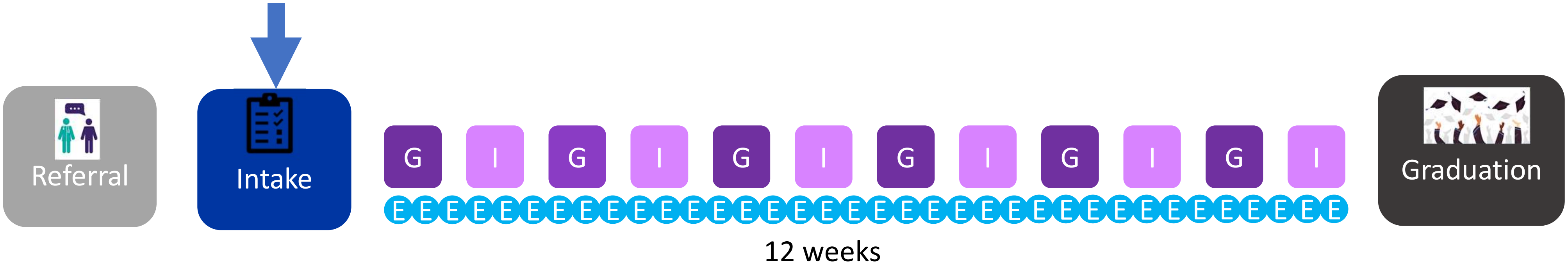

Referral

Intake (in-person)

G = Group Education (in-person)

I = Individual Counseling (in-person or virtual)

E = Exercise (on own or in community)

Graduation (in-person)

Components of Program

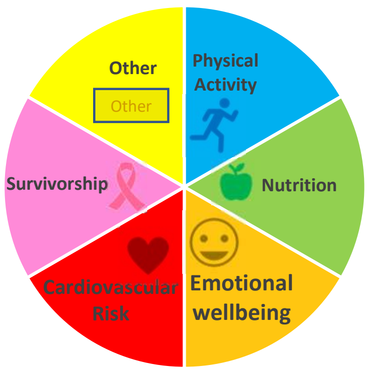

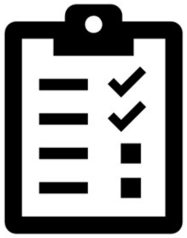

Make Intake Appointment

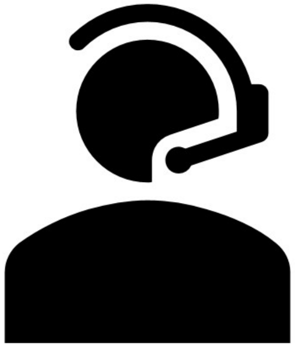

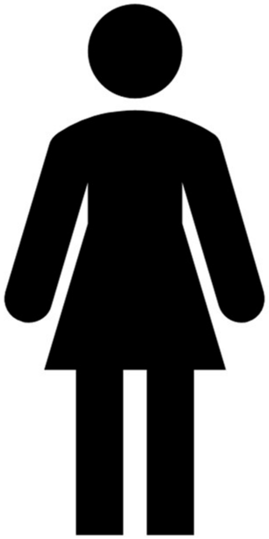

In-person Intake Visit with Nurse (RN) or Exercise Physiologist (EP)

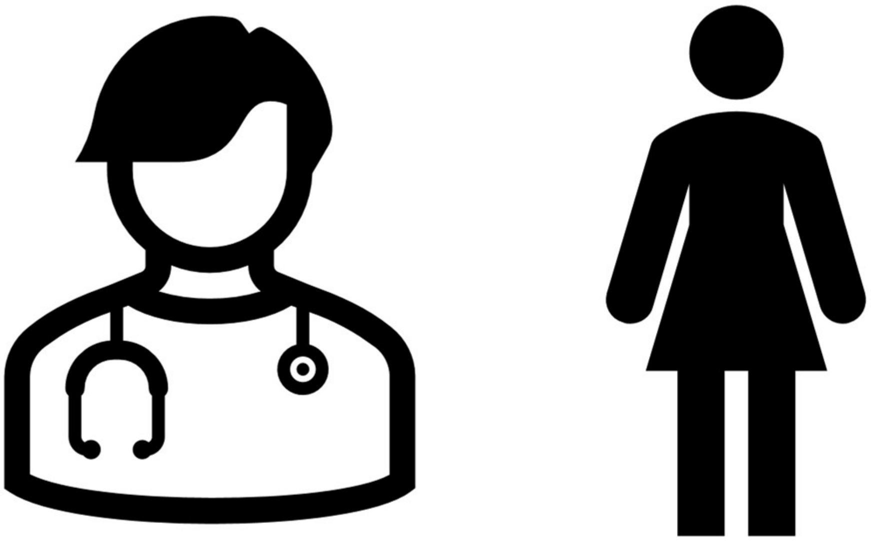

- Questionnaires
- Vitals/Labs
- Six-minute walk
- Goal-setting

Individualized Treatment Plan

- Physical Activity
- Nutrition
- Emotional wellbeing
- Cardiovascular Risk Factors
- Survivorship
- Other items as needed (tobacco, alcohol, etc.) Other

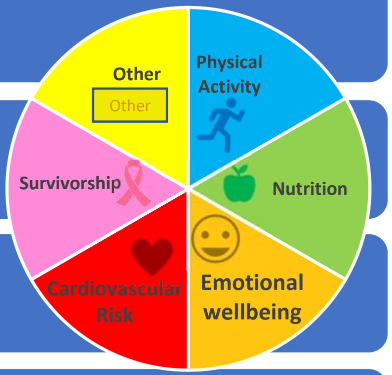

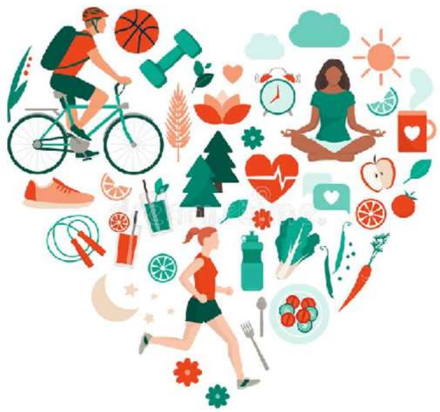

Each item will have an individualized assessment, goal, and plan.

Individualized Treatment Plan - Example

Physical Activity

Assessment
- Questions about current physical activities
- Six minute walk test (walk for six minutes and measure distance)

Goal
- Safely increase exercise intensity and duration

Example Plan
- Exercise prescription example:
  - Warm up: 20 minutes light activity (walking dog)
  - Exercise: After walking dog, walk 10 more minutes at somewhat hard exertion (or to a heart rate goal) every day

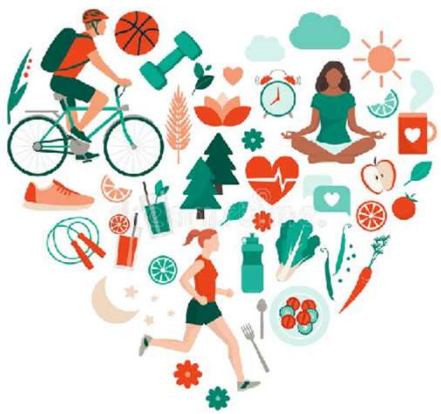

Each item will have an individualized assessment, goal, and plan.

Overall Map

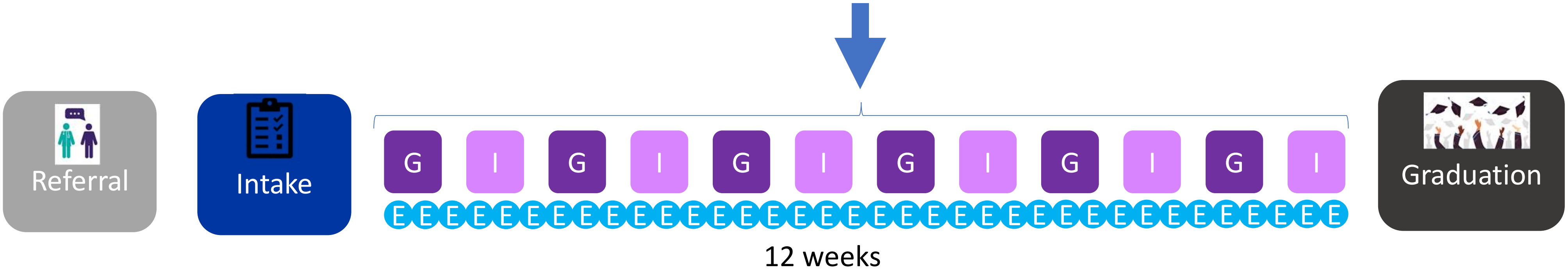

Components of Program

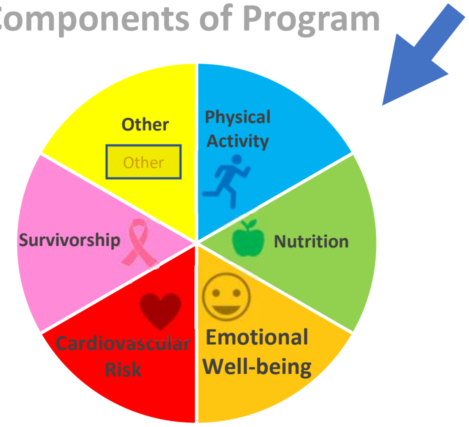

Referral
Intake (in-person)
G = Group Education (in-person)
I = Individual Counseling (in-person or virtual)
E = Exercise (on own or in community)
Graduation (in-person)

G Group Sessions

- Survivorship
- Heart Health
- Physical Activity
- Nutrition
- Emotional wellbeing
- Making and Maintaining Positive Changes

All sessions will include Physical activity, Nutrition, Wellness, and at least 1 other topic.

- Health education by professional
- Peer discussion among group
- Language concordant

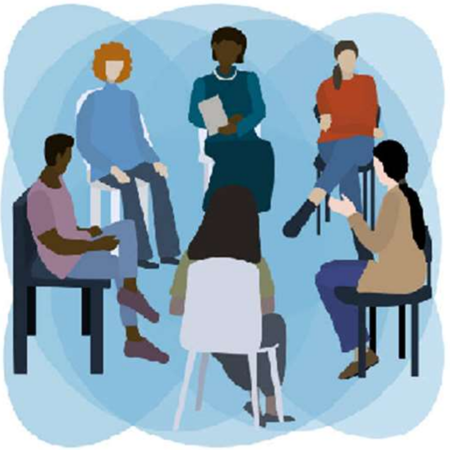

Individual Sessions

- Review progress towards goals
- Update exercise prescription
- Make plan for next two weeks
- Can be in-person or virtual

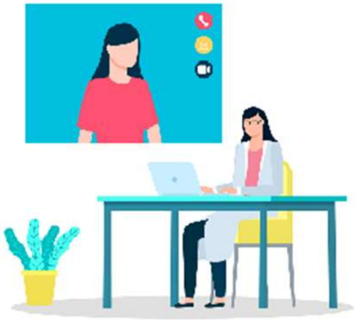

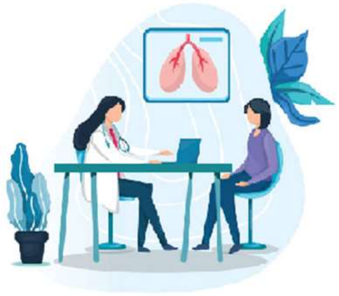

- Health education and counseling by professional
- Individualized
- Language concordant

Exercise Prescription Principles

Individualized

- Addresses breast-cancer specific issues: residual fatigue, arm range of motion, lymphedema
- Addresses concerns/fears about over-doing it
- Fit into daily life

Progress over time

- Goal: at least 150 minutes/week of moderate intensity + strength training 2/week (US Physical Activity Guidelines)

Will issue an equipment package to patients:

Exercise peddler, dumbbells, FitBit, BP cuff

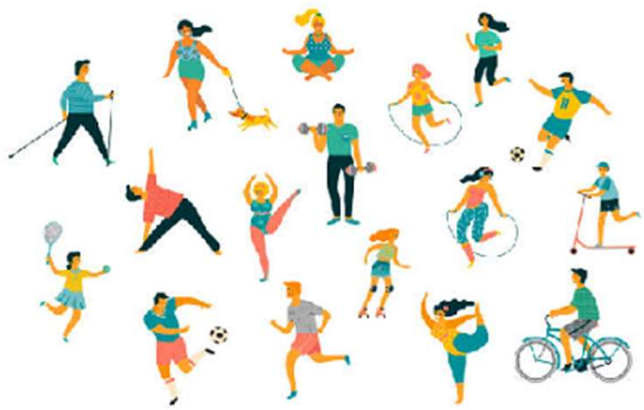

Nutrition Principles

- Education about healthy diet
- Delivered by professional
- Practical
  - Shopping
  - Planning
  - Cooking
- Culturally tailored

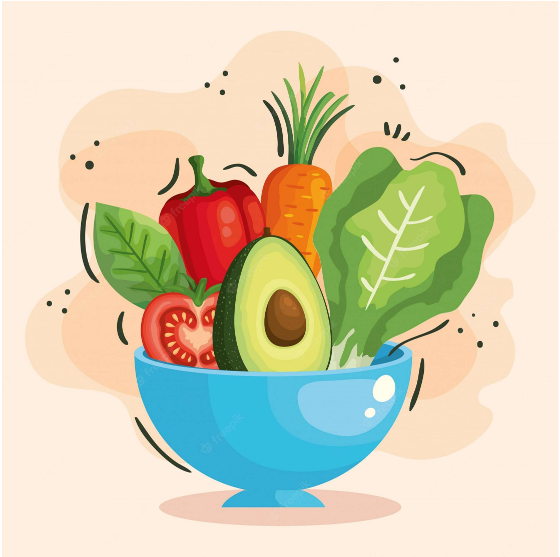

Emotional wellbeing Principles

- Individualized
- Stress management
- Mindfulness not a well-known concept, but may be part of plan
- Sleep
- Positive affect/optimism

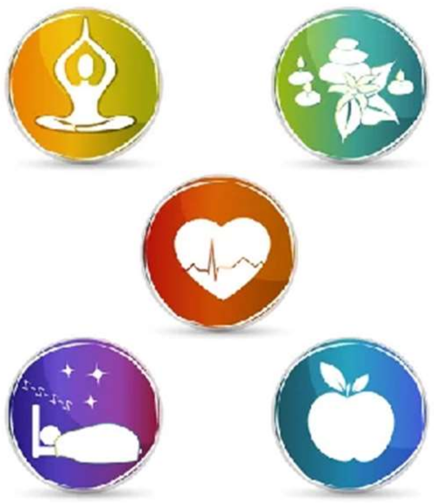

Survivorship Principles

- Return to “normal” life
- Managing long-term side effects
- Body image
- Preventing cancer recurrence
- Support network

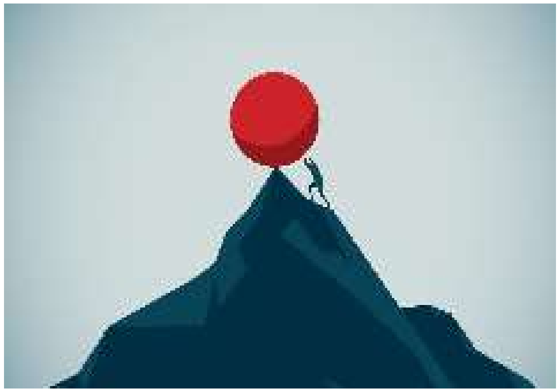

Cardiovascular Risk Factors

- Blood pressure
- Cholesterol
- Diabetes
- Medications and medication adherence
- Weight management

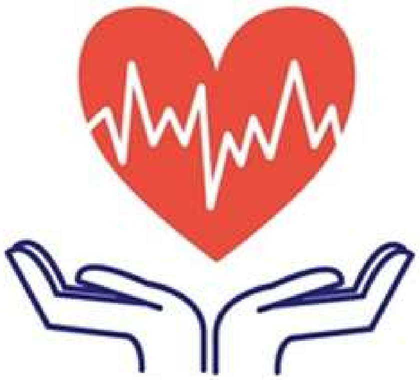

Other

Individualized: Addressing Other Topics

Tobacco/Alcohol/Drugs

- Refer for specialized services as needed

Body Image

Sex & Intimacy

Financial Barriers

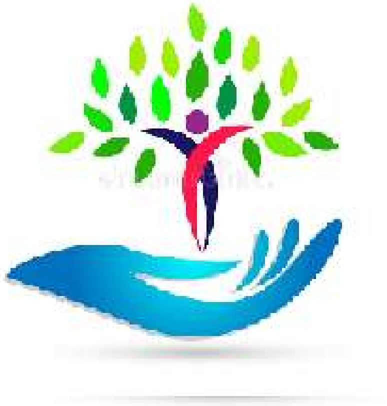

Overall Map

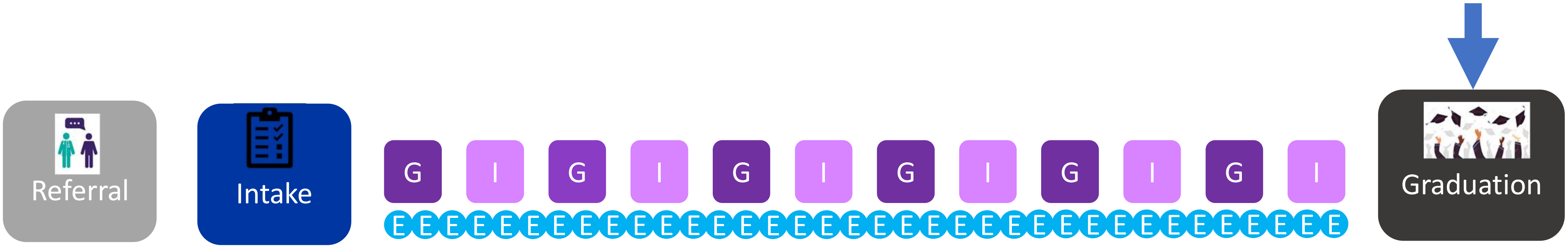

12 weeks

Referral
Intake (in-person)
G = Group Education (in-person)
I = Individual Counseling (in-person or virtual)
E = Exercise (on own or in community)
Graduation (in-person)

Components of Program

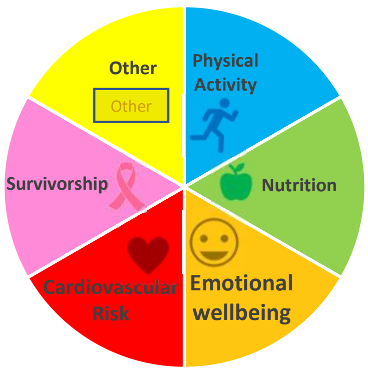

Graduation

- Repeat assessments
- Celebration
- Report of progress
- Long-term plan

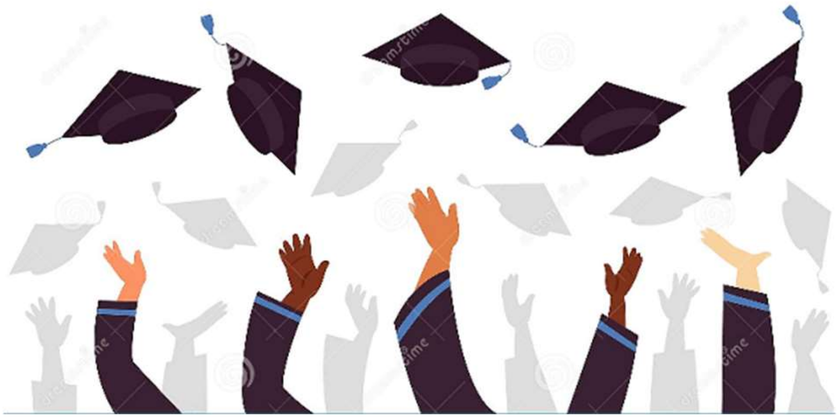

What to name this program

- HEART-ACT (**HEART** Health - **A**fter **C**ancer **T**reatment)
